# Research, Science Identity, and Intent to Pursue a Science Career: A BUILD Intervention Evaluation at CSULB

**DOI:** 10.3390/educsci14060647

**Published:** 2024-06-15

**Authors:** Hector V. Ramos, Kim-Phuong L. Vu

**Affiliations:** 1 BUILD California State University Long Beach and UCLA Diversity Program Consortium, Department of Medicine, University of California, Los Angeles, CA 90095, USA; 2 Psychology Department, California State University, Long Beach, CA 90840, USA

**Keywords:** STEM intervention, research participation, science identity, intent to pursue, BUILD, propensity scores

## Abstract

This paper presents an analysis of survey data to examine the association between participating in one of the National Institute of Health’s (NIH) funded Building Infrastructure Leading to Diversity Initiative (BUILD) program and students’ intent to pursue a career in science. Data were collected from students at California State University Long Beach (CSULB) to examine the effectiveness of the BUILD Scholars program. Both BUILD Scholars and non-BUILD students were surveyed. Propensity score matching was used to generate the non-BUILD comparison group. Multinomial logistic regression results revealed that students participating in the BUILD intervention were associated with significantly higher intent to pursue a career in science. Results also showed the importance of variables such as science identity and research participation when assessing interest in science careers. These findings have implications for STEM program evaluation and practice in higher education.

## 1. Introduction

Recent policy initiatives from the Biden–Harris administration emphasize the importance of strengthening STEM (Science, Technology, Engineering, and Math) education nationwide [1]. For example, the *Raise the Bar: STEM Excellence for All Students* initiative attempts to strengthen STEM education for students and instructors, from pre-k to higher education [1]. Given the objectives of the federal government to improve STEM learning and maintain student interest and pursuit of STEM careers, research is needed to identify evidence-based practices that can be adopted to achieve such goals.

Although STEM degree attainment for underrepresented students has increased over the last 20 years, disparities and barriers continue to persist in STEM careers [2]. For example, Black and Hispanic workers represent only 7% and 8% of all STEM workers, and they have significantly lower salaries than their White and Asian counterparts [2]. Given these barriers for underrepresented students, it is necessary to conduct a review and evaluation of the current STEM intervention landscape. Current research on (STEM) interventions for diverse student populations from various racial/ethnic and socioeconomic backgrounds show that undergraduate research, mentoring, and science identity are beneficial to student academic and career success [3–5].

The Building Infrastructure Leading to Diversity (BUILD) initiative was a national effort to bring together a diverse set of universities to evaluate the effectiveness of different combinations of high-impact practices and innovative STEM interventions to diversify the biomedical and behavioral health sciences workforce [6]. Each of the ten BUILD undergraduate institutions developed approaches intended to determine the most effective ways to engage students from diverse backgrounds in biomedical and behavioral research, with the long-term goal of preparing an increased number of students to pursue research careers in health-related fields [7]. While studies on the overall consortium are underway, it is necessary for each site to conduct additional research into the effectiveness of their own intervention programs.

Guided by best practices established in the higher education literature, the California State University Long Beach (CSULB) BUILD Scholars program [8] provides various high-impact practices in a structured learning community, along with faculty-mentored research in the trainees’ discipline to increase the students’ pursuit of entry into graduate school. The present study assesses various outcomes for the CSULB BUILD Scholars program, and identifies predictors including BUILD participation, research participation, and science identity. Note that research participation is part of the BUILD intervention (see Section 2.2.2 for additional benefits of BUILD participation). Moreover, this study will identify main components of the BUILD intervention at CSULB that influences students’ intent to pursue a science career. Results from the present study can help policymakers decide where they should target their funding to increase diversity and learning in STEM.

The following research questions guide the analysis:

1. Which demographic and student characteristics predict BUILD scholar selection?
2. Is there an association between BUILD participation and increased intent to pursue a research career?
3. Does research participation and science identity influence intent to pursue graduate education as a pathway to a research career?
4. Does research participation and science identity account for the BUILD effect?

### Literature Review

Current research on STEM interventions identifies undergraduate research as a compulsory component of successful undergraduate education programs [9]. The Council on Undergraduate Research identifies undergraduate research as “a mentored investigation or creative inquiry conducted by undergraduates that seeks to make a scholarly or artistic contribution to knowledge [10]”. Undergraduate research is associated with increased academic achievement and longer-term career success in STEM [4,8,11–13]. For example, using propensity score matching to generate a comparison group, Rodenbusch et al. [14] found that freshman undergraduate students who participated in undergraduate research were more likely to graduate with a STEM degree than those who did not. Other studies have found that undergraduate research has a powerful effect on underrepresented student retention [15–17] and is also associated with increased intent to pursue a science career [18,19]. Furthermore, students involved in undergraduate research have an increased likelihood of enrolling in STEM graduate programs [8,20]. Studies have also shown that research experiences increase science identity—another important predictor for STEM success [4,21].

Science identity also has a strong positive relationship with STEM outcomes. Science identity describes students as viewing themselves and acting as scientists [20]. Based on Carlone and Johnson’s [22] construct, students develop science identity through training, performing, and presenting research, and when receiving recognition for their work (e.g., receiving awards). Science identity predicts a host of academic and professional outcomes, such as entering a science occupation, graduate school enrollment in STEM, and pursuit of a STEM degree [23–25]. Moreover, given the importance of science identity as a predictor of STEM success, more recent studies have looked at science identity as an outcome [26]. For example, Eagan et al. [20] found that BUILD interventions increased science identity in first-year college students across four consortium institutions. Thus, any study assessing STEM interventions should consider science identity in the model.

The present study takes the approach of matching for a student’s science identity using propensity scores and then predicting intent to pursue a science career using science identity. Another important component for success in STEM has been associated with student finances; as CSULB BUILD Scholars receive a stipend, among other financial benefits (e.g., funds for supplies and professional travel), it is necessary to include a financial component to the study. This study also introduces a financial variable in the matching procedure. The higher education literature identifies that students with fewer financial resources face more challenges in completing a STEM degree and going on to graduate school [27,28]. Moreover, students from families with a higher income tend to begin at more selective institutions, which have higher degree attainment rates [19]. Also, the financial status of a student is a powerful predictor for majoring in STEM [19]. Finally, socio-economic status is associated with a host of variables prior to college enrollment, for example, students in secondary school with higher incomes receive necessary assistance with college applications and apply to more colleges [29]. These conditions highlight the importance of socio-economic status when assessing students in higher education. Thus, when assessing and conceptualizing STEM interventions, finances must be considered.

## 2. Materials and Methods

This study uses survey data from both BUILD and non-BUILD students at CSULB. We used propensity scores to create a comparison group among non-BUILD students. Then we tested for differences between students who participated in the CSULB BUILD Scholars program with a matched group of their peers. The following sections provide details on our data source and analysis.

### 2.1. Data and Sample

This study uses survey data collected in the Spring of 2019 from CSULB. Data used are from the Student Annual Follow-up Survey (SAFS), developed by the Coordination and Evaluation Center (CEC) at UCLA. Every spring, the CEC invites undergraduate students (both BUILD and non-BUILD) across the BUILD program sites to complete the SAFS. The SAFS asks students about attitudes, perceptions, and participation in a variety of experiences during their time in college. We used data from the SAFS because they provide students’ perceptions at the end of the academic year. The initial sample size for this study is n = 1574, consisting of both BUILD and non-BUILD students. After matching and trimming, we finished with a sample size of n = 1064. This study is cross-sectional because of sample size limitations due to BUILD Scholars beginning the program in their junior (3rd) year and, thus, the students did not have a baseline survey. As a result of using only cross-sectional data, we used Mason et al.’s [30] matching strategy for cross-sectional data. This study is also limited as it does not include a mentoring variable that captured the amount of mentoring or type of mentoring. The only variable provided in the SAFS was a binary mentor variable asking the student whether they have a senior mentor. We conducted a sensitivity analysis which showed no significance or explanatory power of this variable, so we left the variable out of the model.

### 2.2. Variables

#### 2.2.1. Outcome

Response to the survey item: Intent to Pursue a Science Career. This survey item asked students if they will pursue a career in science on a 5-point scale. Given the unequal distribution of the answers to the survey item, for the present study, this variable was recoded to 3 points to effectively run the multinomial logistic regression models [19]. The following coding was used for the intent to pursue variable: 1 = Will not pursue a career in science, 2 = Will possibly pursue a career in science, 3 = Will Pursue a career in science.

#### 2.2.2. BUILD Participation

We created a BUILD dummy variable for BUILD Scholars who have specific requirements to meet eligibility in the program: full-time college enrollment, BUILD major, graduate degree aspirations, and interest in conducting health-related research in biomedical or behavioral sciences. The CSULB BUILD Scholars program is an intensive, 2-year research training program [8]. It begins in the summer prior to the students’ junior year, where they engage in an 8-week program that introduces students to research through various activities in a learning community. Students also engage in a research project with a faculty mentor in their discipline. The summer program ends with a research symposium where the students present their research project.

During the first academic year, BUILD Scholars continue to meet weekly in a learning community to engage in research and professional development activities (ethics training, CV development, conference presentations, application for summer research experiences, etc.), as well as work with their research mentors for about 15 h per week. During the summer before their senior year, students are encouraged to apply and, if accepted, attend a summer research experience at a Ph.D.-granting university. During their senior year, the learning community prepares students for graduate school applications and engages them in a variety of professional development activities; students continue to work with their faculty mentor on research in their disciplines.

BUILD Scholars receive funds for tuition and fees and a monthly stipend, as well as funds for conference travel and research supplies. As noted, they also receive faculty research mentoring, graduate/near-peer mentoring, professional development and training in cohort-based learning communities, and research training through designated course-work. This study uses the BUILD Scholar as the variable of interest; the BUILD Scholar status is the most rigorous component of the BUILD intervention, which means they receive all the BUILD benefits listed above.

#### 2.2.3. Other Variables

The main explanatory variables for the study include: Gender (1 = Male, 2 = Female); Race (coded as 0 or 1 for Hispanic/Latinx, 0 or 1 for Black, 0 or 1 for Asian, 0 or 1 for White, and 0 or 1 for other/multi-racial); Science Identity on a scale from 33–71; Major (coded 0 or 1 for Biomedical, 0 or 1 for Biomedical Social Behavioral, and 0 or 1 for Non-Biomedical); Financial Concerns (also dummy coded 0 or 1 for Some Concerns, 0 or 1 for Serious Concerns, 0 or 1 for No Answer, and 0 or 1 for No Financial Concerns); Degree Aspirations (coded 0 or 1 for MD/PhD/JD, 0 or 1 for Master’s, and 0 or 1 for Bachelor’s); and finally, Research Participation (coded as 1 = Yes 0 = No).

### 2.3. Analyses

#### 2.3.1. Descriptive Analyses

We first conducted descriptive analyses comparing the BUILD Scholars and non-BUILD students on various demographic and background variables. We present the percentages for categorical variables, and the mean and standard deviation for science identity, which is a continuous variable (see Table 1). The differences between the BUILD and non-BUILD students were tested using Chi-square tests and two-sample *t*-tests. As noted above, *Intent to Pursue* is a 3-item categorical variable. We used multinomial logistic regression to fit the data. First, an unweighted multinomial logistic regression model was fit to the data and, after yielding propensity scores and creating balanced groups, a weighted multinomial logistic regression model was fit using the propensity score weights. For all models, the covariates were added in a stepwise fashion.

#### 2.3.2. Propensity Score Modeling

To conduct the propensity score matching, we used a logistic regression model. Logistic regression is ubiquitous in estimating propensity scores [20,31]. This study used SPSS statistical software 29: to account for selection bias, we used demographic variables to help create similar groups. We controlled for race, gender, science identity, income concerns, degree aspirations, and major, and then ran a logistic regression predicting BUILD scholar participation. This logistic regression model yielded propensity scores for each student. Once we had analyzed the model, we matched the BUILD and non-BUILD groups based on propensity score cut-offs. Students from the non-BUILD group with a propensity score lower than those from the BUILD group were cut from the analyses. We yielded a sample size of (101) BUILD Scholars and (1064) students with similar characteristics in the non-BUILD group. BUILD Scholars and their peers were thus non-randomly assigned to either the BUILD group or non-BUILD group but were matched using the propensity scores.

#### 2.3.3. Multinomial Logistic Regression

After conducting the propensity scoring and assigning students to a BUILD group and a non-BUILD group designation, we calculated the Average Treatment Effect (ATE) and the Average Treatment Effect on the Treated (ATT). The ATE weight can be interpreted as the overall effect of the BUILD Scholar experience on a sample of eligible students. In contrast, the ATT weight describes the effect of becoming a BUILD Scholar among research participants with a high probability of receiving the treatment. Subsequently, we ran a multinomial logistic regression model to predict intent to pursue a science career, with BUILD participation as the main variable of interest. Multinomial logistic regression is appropriate for this study because we are not using institutional characteristics and we have a categorical dependent variable with more than two items. We interpreted three models: unweighted, weighted with ATE, and weighted with ATT in our preliminary analyses. In accordance with the objectives of this study, the ATE weight is reported in the results section. After controlling for BUILD participation, research participation, and science identity, the models predicted intent to pursue a science career and are discussed in the results section.

## 3. Results

### 3.1. Descriptive Analyses

Table 1 describes the data for the BUILD and non-BUILD students across the various background characteristics. Comparisons of BUILD Scholars to non-BUILD students showed BUILD Scholars had a higher intent to pursue a science career. For those students that will pursue a science career, the percentage distribution for BUILD Scholars was 88.2% compared to non-BUILD students at 58.3% pre weighting. Next, BUILD Scholars were more likely to report pursuing an MD/Ph.D./JD degree (82% BUILD vs. 26.8% non-BUILD). BUILD Scholars were also more likely to report a “biomedical major” than the non-BUILD students. Moreover, students in the BUILD Scholars identified as women more often than men and were less likely to be Asian than non-BUILD students. There was also a higher percentage of Hispanic and Black students in the BUILD Scholars group than in the non-BUILD group. Finally, science identity scores for BUILD Scholars were higher than for non-BUILD students. BUILD Scholars had a higher science identity score, with a mean of 61.3 vs. a non-BUILD mean of 59.1.

### 3.2. Propensity Scoring

This study used propensity scores to control for selection effects based on the differences identified in descriptive data. We were thus able to control for covariates known to differ between BUILD Scholars and non-BUILD students. Table 2 shows the logistic regression predicting selection as a BUILD Scholar. The most important predictors of participation in the BUILD Scholars program were having a higher science identity and aspiring to an MD/Ph.D./JD degree. In fact, those students with a higher science identity were significantly more likely to select into BUILD, and students that are in pursuit of a PhD/MD/JD degree were more likely to select into BUILD, with an Exp(B) score of 10.457. As BUILD’s goal is to train students that want to have a research career in health-related biomedical and behavioral sciences, it is necessary to account for this criterion when selecting groups. Table 1 presents the covariates before and after weighting and provides the bias reduction calculations.

### 3.3. Outcome Modeling

Table 3 presents the results of the weighted multinomial logistic regression model examining the relationship between participation in the BUILD Scholars program and intent to pursue a career in science. Given the current research landscape and the CSULB BUILD’s objectives, this study reports the ATE weighted model. Two important predictors of intent to pursue a science career are science identity and research participation. Both science identity and research participation contribute to BUILD. Research participation is part of the BUILD intervention, so there should be a decline in the BUILD Scholar effect when research participation is introduced into the model. Also, because BUILD Scholars have higher science identities, the effect of BUILD should also decline when science identity is introduced into the model. To account for these components of the BUILD intervention, the model is presented stepwise, to show how much of the BUILD effect is accounted for when considering research participation and science identity. These variables are accounted for in Mod. 2 and Mod. 3 of Table 3. For example, when we introduced research participation into the model, it took away a large percentage of the BUILD effect for those that will pursue a science career, reducing the odds ratio from 5.9 to 4.156. This happened because research participation is a major component of BUILD.

First, BUILD participation was introduced. Table 3 shows that BUILD Scholars have significantly higher odds ratio of pursuing a career in science than non-BUILD students (exp (B) 5.9, *p* < 0.001). The next step (Mod. 2) introduced research participation to the model and the BUILD effect was effectively cut by more than 20% (exp (B) 4.156, *p* < 0.001) and research participation significantly predicted pursuing a science career (exp (B) 2.491, *p* < 0.001). As noted, the result of this large drop in the BUILD effect is expected as research participation is a strong predictor of intent to pursue a science and a major component of the BUILD intervention [18,19].

Finally, the study controlled for science identity. Once again, there was a decline of over 30% in the BUILD effect, from an odds ratio of 4.156 to 2.964. This result clearly shows that science identity takes up a large portion of the BUILD effect. Science identity also predicts intent to pursue a science career with an (exp (B) of 1.253, *p* < 0.001). These results are consistent with the literature in higher education and the composition of the BUILD intervention because BUILD Scholars have higher science identities [20]. This study also assessed students who will possibly pursue a science career, and the results were not as strong as those who will pursue a science career. Science identity and research participation are both positive significant predictors for possibly pursuing a career in science, but BUILD does not significantly predict possible pursuit of a science career.

Next, it is important to note that we applied the ATE weights rather than the ATT weights. The purpose of focusing on the ATE weights rather than the ATT weights is because as the model progressed, it became apparent that focusing only on those students with almost identical characteristics may not necessarily capture the effect and objectives of the BUILD program. For example, because the ATE weight positively predicts BUILD, it is noteworthy that the BUILD Scholars program increases intent to pursue careers in science among students that may not have as high a science identity or other important characteristics. Thus, studying the overall treatment effect can help target those students that the BUILD Scholars program may benefit to a greater degree [20]. Therefore, this study’s results provide insight into the current higher education literature.

### 3.4. Limitations

This study’s limitations result from a shortage of longitudinal data for the CSULB site. This study used a cross-sectional analysis. As most of the students began the BUILD program in their junior year, and the survey was administered at the end of their junior year, it is not possible to establish a baseline for a longitudinal analysis. Next, there is a small number of BUILD Scholars overall, so sample size limitations begin to arise. Another limitation of this study is the use of indicator flags for missing data rather than multiple imputation. Given that the data were not missing at random, the use of multiple imputation was not possible; therefore, we created indicator flags during propensity scoring to retain as many cases as possible. Although a longitudinal study and multiple imputation would be optimal to establish causal inference, this study provided a quasi-experimental research design that can provide strong association from the various analyses.

## 4. Discussion

This study aimed to predict whether the intent to pursue a science career for BUILD Scholars is higher when compared to similar (i.e., matched) students without the BUILD intervention. Results showed a relationship between the BUILD intervention and the selection criteria consistent with the higher education literature: students with higher science identity were more likely to select into the BUILD Scholars program, a result consistent with Eagan et al. [20]. This study therefore confirms the overwhelming literature on science identity as a positive predictor for a variety of student outcomes, and this specific paper identifies the importance of science identity in selecting into a STEM intervention program and predicting intent to pursue a career in science.

Next, the ATE weighted multinomial logistic regression suggests that participation in the BUILD Scholars program is associated with higher likelihood of pursuing a science career. The model shows that the BUILD effect was significant throughout each step; also, the introduction of science identity and research participation into the model explained a high percentage of the variation of the BUILD effect because of a drop in the odds ratios when the variables entered the model. Thus, the BUILD Scholars effect remains significant throughout the entire model, but the effect size is greatly reduced when research participation is considered and then reduced further when science identity is introduced.

### 4.1. Implications for Future Research

Future research can begin to assess STEM interventions longitudinally on a large scale. For example, researchers can use national survey data to conduct quasi-experimental research on students that have been involved in STEM intervention programs, then they can compare the outcomes of those programs over time and identify the criteria that makes them successful or not. Next, researchers can conduct mixed method studies that will show an association between the qualitative and quantitative data, thus strengthening the argument of whether the intervention succeed in increasing students’ pursuit of a science career.

Finally, future researchers can examine prior studies with new research to update the literature. This is critically important, as it is possible that the higher education atmosphere has changed significantly over the years. For example, over the last twenty years, significant strides have been undertaken to ameliorate the lack of diversity in STEM disciplines [16]. Hopefully, new research will show changes in outcomes from previous studies. Several researchers have already used new datasets to either confirm or show improvement in a host of STEM outcomes over time [19,25,29]. Prodigiously, some of this research has shown improvement for diverse student populations, yet additional improvement is still necessary.

### 4.2. Implications for Future POLICY

This study has implications for future policy. First, it is necessary to continue to fund comprehensive research interventions such as BUILD; these initiatives include a host of best practices that improve student experiences and outcomes, both career and academic. Second, because this study highlights the various components of the BUILD intervention that are effective, policymakers can begin to fund and implement these types of programs. For example, this study shows that there is a strong association between research participation and intent to pursue a science career. Policymakers can therefore increase funding for programs that include STEM research in higher education.

## Figures and Tables

**Table 1.** Descriptive Statistics of the Sample Before and After Weighting.

Variable	BUILD Scholars			Control Group—Pre-Weighting			Control Group—Post-Weighting			
	%	Mean	SD	%	Mean	SD	%	Mean	SD	Bias Red.
**Intent to Pursue**										
Definitely Will Not Pursue	5.2			20.1			9.7			0.69
Maybe Will Pursue	6.6			21.6			11.0			0.70
Defintitely Will Pursue	88.2			58.3			79.4			0.71
**Gender**										
Female	68.7			65.8			66.8			0.34
Male	29.3			33.9			32.9			0.21
Other Category	2.0			N/A			N/A			N/A
**Race/Ethnicity**										
Race: Latinx	49.2			48.2			50.5			0.43
Race: Black/African American	5.1			4.1			5.7			0.62
Race: Asian	29			33.5			31.1			0.53
Race: White	13			11.3			9.2			0.81
Race: Other Categories	3.03			2.99			3.4			0.09
**Degree Aspiration**										
Bachelor’s Degree	7			32.7			7.4			0.98
Master’s Degree	9			31.8			7.3			0.93
MD/PhD/JD	82			26.8			83.2			0.98
Other	N/A			8.6			N/A			N/A
Science Identity		61.31	6.2		59.1	6.0		59.5	6.5	0.82
**Major**										
Biomed	68.2			58.1			72.6			0.70
Social Behavioral	20.5			14			17.0			0.46
Non-Biomed	11.4			27.9			10.4			0.97
**Financial Worry**										
Choose not to answer	2.1			1.9			1.9			N/A
Major (not sure if I will have enough funds to complete college)	17.7			19.6			19.0			0.32
Non (I am confident that I will have enough funds to complete college)	17.7			21.2			19.3			0.54
Some (I am confident that I will have sufficient funds to complete college)	62.5			57.3			59.8			0.48

**Table 2.** Results of Binary Logistic Regression Predicting BUILD Scholar Selection.

	B	S.E.	Sig.	Exp(B)
Degree Aspiration MA/MS	1.215	1.139	0.286	3.370
Degree Aspiration other	−0.168	0.462	0.716	0.845
Degree Aspiration PhD/MD/JD	2.347	0.360	0.000 ***	10.457
Female	−0.119	0.267	0.657	0.888
Science Identity	0.134	0.019	0.000 ***	1.144
Latino	0.179	0.374	0.633	1.196
Black	0.752	0.669	0.261	2.121
Asian	−0.123	0.395	0.755	0.884
Other/multi-racial	−1.068	0.732	0.144	0.344
Major natural Science	−0.252	0.398	0.527	0.777
Major social behavioral	0.408	0.450	0.365	1.504
Noanswerincome concerns	−0.944	0.841	0.262	0.389
Some income concerns	0.010	0.303	0.973	1.010
Serious income concerns	−0.002	0.385	0.996	0.998
Constant	−11.226	1.191	0.000	0.000

*** *p* < 0.001.

**Table 3.** Results of Multinomial Logistic Regression on Intent to Pursue (ATE Weight).

Independent Variables	Mod. 1			Mod. 2			Mod. 3		
Possibly Pursue	B	SE	Exp(B)	B	SE	Exp(B)	B	SE	Exp(B)
Constant	0.068	0.091		−0.870	0.160		−4.284	0.963	
BUILD Scholar	0.175	0.206	1.19	−0.070	0.213	0.743	−2.30	0.219	0.794
Research Participation				0.759	0.109	2.136 ***	0.739	0.109	2.094 ***
Science Identity							0.066	0.018	1.069 ***
Pursue	B	SE	Exp(B)	B	SE	Exp(B)	B	SE	Exp(B).
Constant	1.063	0.076		−107	0.133		−12.169	0.929	
BUILD Scholar	1.775	0.161	5.9 ***	1.425	0.166	4.156 ***	1.087	0.176	2.964 ***
Research Participation				0.913	0.094	2.491 ***	0.761	0.097	2.141 ***
Science Identity							0.225	0.017	1.253 ***

*** *p* < 0.001.

## Data Availability

The original contributions presented in the study are included in the article, further inquiries can be directed to the corresponding author.
